# Integrating Conventional and Emerging Approaches in Cardiovascular Risk Assessment and Management

**DOI:** 10.1002/puh2.70378

**Published:** 2026-09-15

**Authors:** Sana Rasheed, Azka Mujeeb, Misbah Sarfraz, Abedin Samadi, Ahmed Asad Raza

**Affiliations:** ^1^ Jinnah Sindh Medical University (SMC) Karachi Pakistan; ^2^ Kabul University of Medical Sciences Abu Ali Sina Kabul Afghanistan

**Keywords:** cardiovascular disease prevention, cardiovascular risk assessment, health equity, implementation, precision medicine, risk stratification

## Abstract

Cardiovascular diseases (CVDs) remain the leading cause of global mortality, and the burden is rising fastest in low‐ and middle‐income countries (LMICs). Conventional risk calculators, such as Framingham and Systematic Coronary Risk Evaluation (SCORE), incorporate age, blood pressure, cholesterol, diabetes and smoking, yet they underperform in populations that differ from their derivation cohorts and leave substantial residual risk unexplained. This narrative review evaluates traditional risk factors alongside emerging tools, including coronary artery calcium (CAC) scoring, polygenic risk scores (PRS) and inflammatory biomarkers such as high‐sensitivity C‐reactive protein (hsCRP) and interleukin‐6. It examines the contribution of lifestyle and environmental exposures—chronic stress, physical inactivity, and air pollution—to inflammatory and atherogenic pathways and considers the role of imaging, molecular biomarkers, and artificial intelligence (AI)‐assisted algorithms in risk stratification. Management strategies combining pharmacological therapy (statins, antihypertensives and antidiabetic agents) with individualised de‐escalation, lifestyle modification and precision medicine are summarised. Rather than describing each tool in isolation, this review compares them directly on incremental predictive value, cost, infrastructure requirements, equity implications and readiness for routine implementation, and proposes a stepwise pathway for integrating them into cardiovascular risk assessment. Particular attention is given to feasibility in resource‐constrained health systems, where most of the global CVD burden now falls. We conclude that the near‐term public health gain lies less in broad adoption of novel tests than in their selective use where they change management, coupled with stronger delivery of established, low‐cost preventive care.

## Graphical abstract

Conventional risk factors (hypertension, dyslipidaemia, diabetes, smoking, obesity and physical inactivity) account for roughly 70% of population‐attributable cardiovascular risk and anchor a proposed three‐tier pathway: universal low‐cost assessment (Tier 1), selective testing such as hsCRP or PRS where it would change management (Tier 2) and resource‐intensive tools such as CAC scoring or AI‐assisted algorithms reserved for defined clinical uncertainty (Tier 3, not yet routine). Management combines lifestyle modification, pharmacological therapy and emerging precision‐medicine approaches, with equitable implementation requiring recalibration of tools largely derived from high‐income, European‐ancestry cohorts.

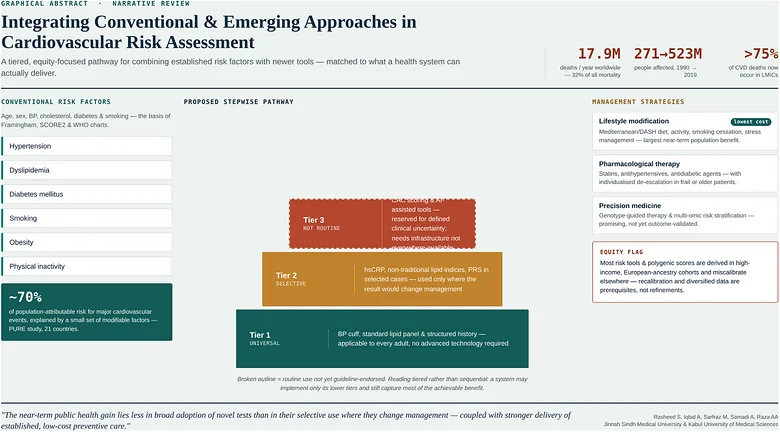

## 1 Introduction

Cardiovascular diseases (CVDs) are the number one cause of death worldwide, accounting for about 17.9 million deaths annually, which is 32% of mortality worldwide [1]. The Global Burden of Disease Study 2019 documented an almost doubling in CVD burden from 271 million in 1990 to more than 523 million in 2019, with disproportionate distribution in low‐ and middle‐income countries (LMICs) [2]. In spite of advances in therapeutics, early detection and prevention of CVD are vital in minimising morbidity and mortality [3]. Because more than three quarters of CVD deaths now occur in LMICs, the practical value of any risk assessment strategy depends as much on whether it can be delivered in resource‐constrained settings as on its statistical performance [4].

Traditional risk assessment calculators like the Framingham Risk Score and the Systematic Coronary Risk Evaluation (SCORE) have gained widespread use in estimating 10‐year cardiovascular risk [5, 6]. These calculators are premised on the classic risk factors of age, sex, blood pressure, cholesterol, diabetes and smoking. Their predictive capability, however, differs markedly by age, race and sex, frequently resulting in misclassification of risk [7, 8, 9]. This is in part because most were derived in predominantly high‐income, European‐ancestry cohorts and require recalibration before use elsewhere; the revised World Health Organization (WHO) risk charts and SCORE2 were developed specifically to address regional miscalibration [10, 11]. Additionally, such models do not include new risk determinants, including genetic susceptibility, inflammatory biomarkers and socio‐environmental factors [12].

New technologies, such as coronary artery calcium (CAC) scoring, polygenic risk scores (PRS) and artificial intelligence (AI)‐augmented algorithms, have been promising in further defining risk stratification beyond traditional models [13, 14]. CAC scoring, in fact, has provided enhanced discrimination among intermediate‐risk patients, whereas PRS potentially advances risk prediction in the young and those with a strong family history of early‐onset CVD [14].

**FIGURE 1:**
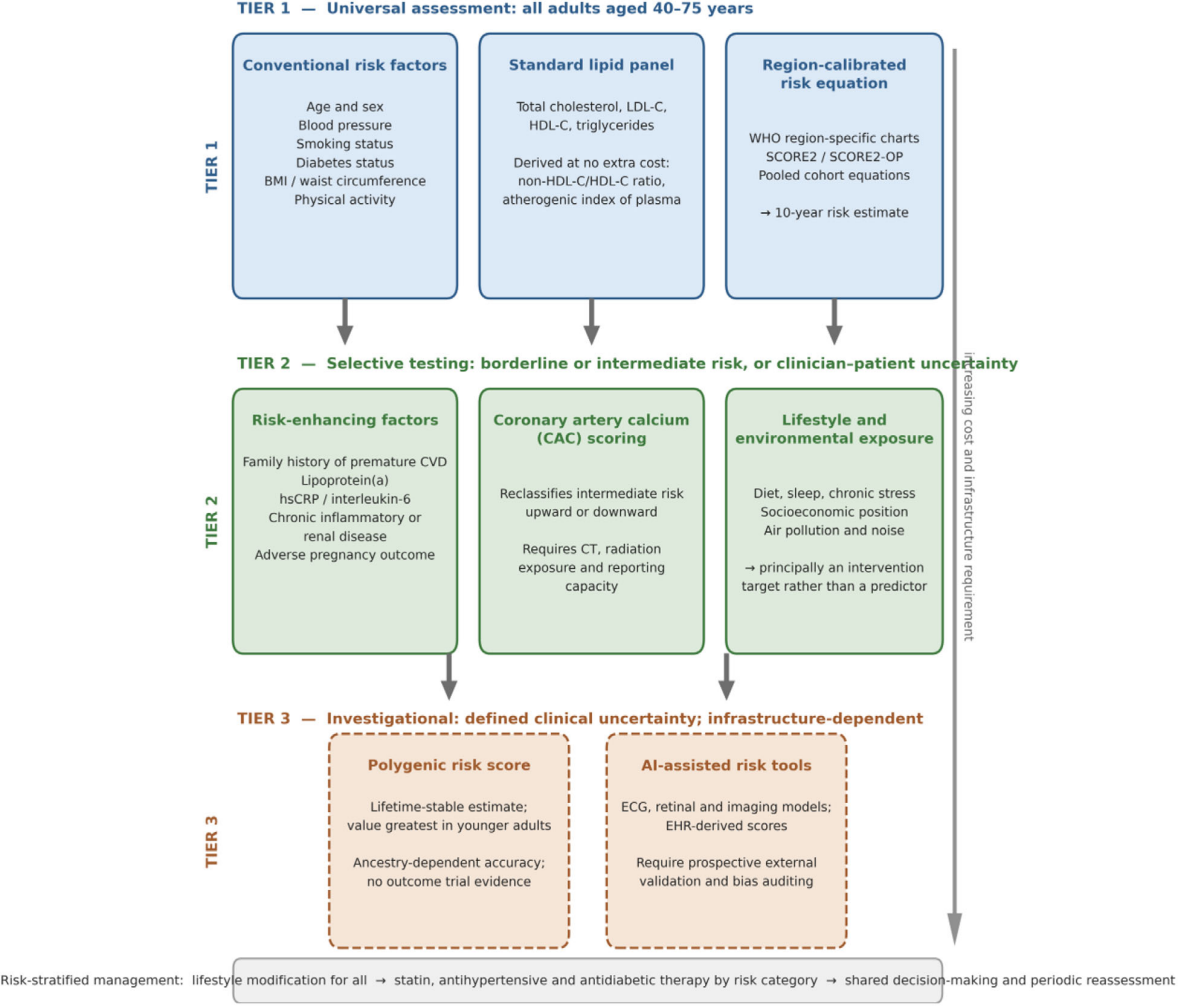
Proposed stepwise pathway for integrating conventional and emerging approaches to cardiovascular risk assessment. Tier 1 is applicable to all adults and requires no technology beyond a blood pressure cuff, a standard lipid panel and a structured history. Tier 2 is applied selectively where the result would change management. Tier 3 is reserved for defined clinical uncertainty and presupposes infrastructure that is not universally available. Broken outlines indicate components whose routine use is not currently guideline‐endorsed. Arrows on the right indicate the direction of increasing cost and infrastructure requirement and of decreasing population reach.

Existing reviews and guideline documents describe these tools individually and in considerable depth. What is addressed less often is how they compare with one another when judged against the criteria that determine population‐level impact: incremental predictive value over established models, unit cost, infrastructure and workforce requirements, effect on existing inequities and readiness for routine use. This review therefore has three aims. First, to summarise conventional and emerging approaches to cardiovascular risk assessment and management. Second, to compare those approaches explicitly on implementation criteria rather than on discriminative performance alone (Table 1). Third, to propose a stepwise pathway (Figure 1) indicating where each tool might reasonably sit in a risk assessment sequence and to consider how such a pathway could be adapted to health systems of differing capacity. We do not claim an exhaustive account of any individual technology; the intention is to make explicit the trade‐offs that determine whether these tools can be deployed equitably at scale.

**TABLE 1:** Comparative appraisal of emerging cardiovascular risk assessment tools.

Tool	Incremental predictive value	Cost and infrastructure	Equity implications	Implementation readiness
Coronary artery calcium (CAC) scoring	Substantial in intermediate‐risk adults; reclassifies a meaningful proportion up or down [13, 15]	Moderate per scan; requires CT scanner, radiation exposure and trained reporting	Concentrated in urban tertiary centres; usually out‐of‐pocket in systems without universal coverage	High in well‐resourced settings for defined indications; low where CT access is limited
Polygenic risk scores (PRS)	Modest once clinical risk factors are known; greatest in younger adults and those with early family history [16, 17]	Falling unit cost; one‐off test but requires genotyping platform, bioinformatics and counselling capacity	Accuracy is ancestry‐dependent; risks widening disparities without diversified reference data [18]	Not currently guideline‐endorsed for routine use; no outcome trial evidence
Inflammatory biomarkers (hsCRP, IL‐6)	Small; change in *C* statistic typically <0.01 when added to established models [19, 20]	Low; assays run on existing laboratory platforms	Broadly accessible where basic laboratory services exist	Available now; clearest role in selecting patients for anti‐inflammatory therapy rather than primary risk estimation
Non‐traditional lipid indices (non‐HDL‐C/HDL‐C, AIP, Castelli indices)	Moderate; outperform conventional lipids for identifying high‐risk individuals in some populations [21]	None beyond a standard lipid panel; derived by calculation	Most equitable of the options considered; deployable wherever lipids are measured	Immediately deployable; requires only a change in reporting convention
AI‐assisted risk tools	Promising but heterogeneous; most evidence is retrospective and internally validated [22]	Low marginal cost per patient once deployed; high dependence on digital infrastructure and technical support	Can extend reach through task‐shifting, but algorithmic bias may disadvantage under‐represented groups [22]	Early; prospective external validation and bias auditing required before routine use

Abbreviations: AI, artificial intelligence; HDL‐C, high‐density lipoprotein cholesterol; hsCRP, high‐sensitivity C‐reactive protein; IL‐6, interleukin‐6.

## 2 Conventional Cardiovascular Risk Factors

According to the guidelines presented by the American Heart Association regarding risk assessment and prevention of atherosclerotic cardiovascular disease (ASCVD), several risk factors are well‐established determinants of ASCVD. They include hypertension, dyslipidaemia, diabetes mellitus, smoking, obesity and physical inactivity [8, 23].

### 2.1 Hypertension

Elevated blood pressure is the major independent modifiable risk factor for CVD [23]. In a meta‐analysis of individual data from one million adults in 61 prospective studies, the risk of vascular mortality increased in a log‐linear fashion from systolic blood pressure (SBP) levels below 115 to above 180 mmHg and from diastolic blood pressure (DBP) levels below 75 to above 105 mmHg. A 20 mmHg higher SBP and a 10 mmHg higher DBP were each associated with at least a twofold increase in mortality from stroke, ischaemic heart disease and other vascular disease [24].

### 2.2 Dyslipidaemia

Dyslipidaemia refers to abnormal lipid profiles, particularly elevated serum low‐density lipoprotein cholesterol (LDL‐C) or total cholesterol and is a key modifiable risk factor for ASCVD [23]. The atherogenic pattern comprises elevated LDL‐C, triglycerides and lipoprotein(a), with reduced high‐density lipoprotein cholesterol (HDL‐C) [25].

### 2.3 Diabetes Mellitus

Diabetes mellitus increases CVD risk through multiple metabolic pathways and is included in all major risk assessment tools [23]. It carries both microvascular and macrovascular complications, and CVD is the leading cause of mortality in this group; a systematic review of 57 studies, including more than 4.5 million people with Type 2 diabetes, found that approximately one third had established CVD [26].

### 2.4 Smoking

Smoking is a modifiable risk factor related to premature cardiac events [23]. Through several pathogenic mechanisms, its cardiovascular effects include coronary artery disease, acute coronary syndrome, atrial fibrillation, venous thromboembolism and an increased incidence of stroke and of hospital admission for heart failure [27].

### 2.5 Obesity

Obesity is typically defined as a body mass index (BMI) ≥30.0 kg/m^2^ and confers high risk for Type 2 diabetes, hypertension and CVD. Beyond its association with multiple comorbidities, obesity is an independent risk factor for CVD. Its cardiovascular effects include increased adipose tissue perfusion, haemodynamic changes from increased cardiac output, left ventricular diastolic dysfunction, obesity‐related cardiomyopathy, endothelial dysfunction, venous insufficiency and sleep apnoea, which together contribute to acute coronary syndrome, coronary artery disease, pulmonary hypertension and stroke [28].

### 2.6 Physical Inactivity

Physical inactivity, by the threshold defined by the American Heart Association, is engaging in less than 150 min per week of moderate‐intensity aerobic activity or less than 75 min per week of vigorous‐intensity aerobic activity. It is recognised as an independent modifiable risk factor, with sedentary behaviour increasing the risk of ASCVD events [8, 23]. Globally, 7.6% of CVD deaths are attributable to physical inactivity, and 74% of those deaths occur in middle‐income countries [29].

Collectively, these conventional factors remain the dominant contributors to CVD at population level. In the Prospective Urban Rural Epidemiology (PURE) study of 155,722 participants across 21 countries, a small number of modifiable risk factors accounted for approximately 70% of the population attributable risk for major cardiovascular events, with metabolic risk factors predominating overall and household air pollution and poor diet contributing disproportionately in low‐income countries [30]. This has a direct implication for the sections that follow: Emerging tools should be assessed against what could be achieved by fuller delivery of interventions targeting these established factors.

## 3 Emerging Risk Factors

In addition to the traditional cardiovascular risk factors, emerging cardiovascular risk factors include inflammatory markers (e.g., high‐sensitivity C‐reactive protein [hsCRP]), genetic risk scores such as PRS for coronary artery disease and lifestyle and psychosocial influences. These factors contribute to residual cardiovascular risk beyond traditional markers.

### 3.1 Inflammatory Markers

Inflammatory markers, such as hsCRP, reflect systemic inflammation, which plays a central role in atherosclerosis progression and plaque instability. Elevated hsCRP is independently associated with increased cardiovascular events and correlates with lifestyle factors, including smoking, obesity, physical inactivity and poor diet. Although hsCRP can assist risk reclassification, particularly in borderline or intermediate‐risk patients, it is not routinely measured in all asymptomatic individuals because the incremental gain in discrimination is small and the implications for management are often unclear [19, 31, 32, 33].

### 3.2 Genetic Risk Scores

Genetic risk scores, including PRS, capture inherited susceptibility to CVD and particularly to coronary artery disease. Recent large cohort data demonstrate that PRS have a magnitude of risk contribution comparable to traditional factors such as hypertension and dyslipidaemia. However, their incremental value over established clinical risk factors and CAC scoring remains limited in middle‐aged and older adults [31, 34]. A further constraint is that most PRS have been derived in European‐ancestry cohorts and perform substantially less well in other ancestral groups; without deliberate diversification of the underlying genomic datasets, clinical deployment risks widening rather than narrowing existing disparities [18]. This is a material consideration for South Asian, African and Middle Eastern populations, which carry a high CVD burden but are markedly under‐represented in genome‐wide association studies.

### 3.3 Lifestyle and Environmental Exposures

Lifestyle and psychosocial factors such as chronic stress, socioeconomic deprivation, unhealthy diet, insufficient sleep and environmental exposures, including air pollution and noise, modulate cardiovascular risk partly through inflammatory and immune pathways. These factors influence haematopoiesis and innate immunity, promoting chronic vascular inflammation and atherothrombosis [35, 36, 37]. Because several of these exposures are structural rather than individual, they are more amenable to policy‐level than to clinic‐level intervention, which is an argument for treating them as public health targets rather than as additional variables in a risk equation.

Emerging risk factors, such as hsCRP, PRS and psychosocial or lifestyle influences, therefore provide additional insight into cardiovascular risk, particularly residual risk beyond traditional factors. The American Heart Association recognises their potential role in refining risk assessment and guiding preventive strategies in selected patients with uncertain risk profiles [31, 34]. A summary of selected emerging and non‐traditional cardiovascular risk factors, their supporting evidence and clinical applicability is presented in Table 2 [16, 20, 32, 38, 39].

**TABLE 2:** Selected emerging and non‐traditional cardiovascular risk factors: Evidence and clinical relevance.

Risk factor	Evidence level	Clinical relevance	Key sources
High‐sensitivity C‐reactive protein (hsCRP)	Moderate. Meta‐analyses show only a small improvement in discrimination and reclassification when added to established models (change in *C* statistic <0.01), with inconsistent findings across studies	Selectively useful for refining risk in intermediate‐risk patients (10%–20% 10‐year risk); optional, not routine	Pearson et al. [38], Lin et al. [20]
Genetic risk scores (PRS)	Growing. Greatest incremental value in younger individuals and those at borderline or intermediate risk; attenuated once clinical risk factors and CAC are known	Potential precision medicine role in guiding earlier or more intensive prevention; performance is ancestry‐dependent and not yet guideline‐endorsed for routine use	O'Sullivan et al. [16], Phulka et al. [39], Martin et al. [18]
Lifestyle and psychosocial factors	Well established as causal contributors, but addition to formal prediction models yields only modest improvement and is not currently guideline‐recommended for stratification	Strong observational evidence of independent association with cardiovascular risk and inflammatory burden; principal value lies in intervention rather than prediction	Bay et al. [32]

Abbreviations: CAC, coronary artery calcium; PRS, polygenic risk scores.

## 4 Advances in Risk Assessment and Stratification

### 4.1 Novel Risk Prediction Models (PRS)

PRS are emerging as tools to enhance CVD risk prediction by aggregating the effects of numerous genetic variants identified through genome‐wide association studies [14]. A PRS provides a stable lifetime measure of genetic predisposition, allowing risk assessment at any age, including at birth, independent of traditional risk factors [17]. When incorporated into conventional models such as SCORE2 or Framingham, PRS yields modest to moderate improvements in risk discrimination and reclassification, particularly benefiting individuals with borderline or intermediate clinical risk [16, 17]. PRS further contributes additive predictive value beyond family history and can identify high‐risk individuals not captured by traditional algorithms, including younger populations and those with monogenic disease [16, 17]. Although PRS are commercially available and attracting attention, current guidelines do not yet support their widespread clinical use, emphasising the need for further research and careful assessment of utility and implementation challenges [16, 17]. No randomised trial has yet demonstrated that PRS‐guided management improves cardiovascular outcomes, which remains the principal evidential gap.

### 4.2 Imaging Techniques (Coronary Calcium Scoring)

Coronary computed tomography angiography (CCTA) and CAC scoring are pivotal imaging modalities that enhance cardiovascular risk assessment by directly evaluating coronary artery disease and quantifying atherosclerotic burden. CCTA provides high negative predictive value for excluding obstructive coronary disease, whereas CAC scoring independently predicts major adverse cardiovascular events and mortality, often outperforming traditional risk indices such as the Revised Cardiac Risk Index. CAC scoring is simpler and avoids contrast but cannot detect non‐calcified plaque, which highlights the complementary roles of these techniques [40].

The Agatston‐based CAC scoring system classifies patients into risk categories: 0 (no detectable calcified plaque), 1–10 (minimal), 11–100 (mild), 101–400 (moderate), >400 (severe) and >1000 (extensive). CAC scoring adds value beyond traditional tools such as the Framingham Risk Score, especially for reclassifying intermediate‐risk individuals. Guidelines from the ACC/AHA, the Canadian Cardiovascular Society and the European Society of Cardiology endorse CAC scoring for asymptomatic adults at borderline to intermediate risk. AI is increasingly used to improve CAC quantification and workflow efficiency [15].

Recent work emphasises refining CAC scoring by incorporating calcium density, regional distribution and extracoronary calcification. Lower calcium density may signal higher risk, whereas diffuse distribution across vessels indicates worse prognosis. Emerging approaches also suggest integrating parameters such as epicardial fat and liver attenuation to further individualise risk assessment, aligning with precision medicine strategies [41]. The principal constraints are that CAC requires computed tomography with its associated capital cost, ionising radiation exposure and trained reporting capacity; that it detects established rather than incipient disease, limiting its value in younger adults; and that a CAC score of zero has a shorter warranty period in people with diabetes or a strong family history.

### 4.3 Biomarkers Beyond Cholesterol

Traditional lipid markers, such as LDL‐C and HDL‐C, do not fully capture the complexity of CVD risk. Biomarkers beyond cholesterol are gaining attention for their ability to reflect atherosclerotic activity, inflammation, plaque instability and myocardial stress [42].

High‐sensitivity cardiac troponins (hs‐cTn) detect low levels of myocardial injury, even in asymptomatic individuals, improving early diagnosis and risk stratification [17]. Interleukin‐6 (IL‐6) and hsCRP are inflammatory biomarkers linked to cardiovascular events and heart failure progression [42]. Growth differentiation factor‐15 (GDF‐15), a cytokine associated with oxidative stress and ischaemia, predicts adverse cardiovascular outcomes, including mortality and heart failure, though it lacks cardiac specificity. Galectin‐3, a fibrosis‐related marker, contributes to plaque instability and cardiac remodelling, with elevated levels associated with infarct size, heart failure and increased mortality [43].

Lipoprotein‐associated phospholipase A2 (Lp‐PLA2), an enzyme involved in vascular inflammation, is associated with plaque vulnerability and coronary artery disease, with its role depending on whether it is bound to LDL (pro‐inflammatory) or HDL (protective); elevated levels predict cardiovascular events independently of traditional lipids. Osteocalcin contributes to vascular calcification, promotes endothelial repair and is inversely associated with atherosclerotic plaque burden. Angiogenin promotes plaque neovascularisation and instability, with elevated levels linked to coronary artery disease, heart failure and mortality [43]. MicroRNAs (miRNAs) regulate lipid metabolism, inflammation and apoptosis; specific species (miR‐1, miR‐133a, miR‐208a/b and miR‐499) rise rapidly after myocardial infarction and may offer diagnostic value earlier than troponin [42, 43].

Non‐traditional lipid parameters also offer predictive value. Residual lipoprotein cholesterol, the non‐HDL‐C/HDL‐C ratio, Castelli's risk indices (CRI‐I and CRI‐II), and the atherogenic index of plasma are associated with carotid plaque vulnerability and stenosis severity, and outperform conventional lipids in identifying high‐risk individuals, particularly in patients with acute ischaemic stroke [21]. These indices are derived by calculation from a standard lipid panel and therefore carry no additional laboratory cost, which makes them the most immediately deployable of the markers discussed here.

Collectively, these biomarkers reflect multiple pathophysiological pathways and may improve early diagnosis and individualised risk prediction. It should be noted, however, that few have been shown in prospective studies to alter management or improve outcomes, and none is currently recommended for routine population screening.

### 4.4 Comparative Appraisal of Emerging Approaches

Considered together, these approaches differ less in whether they add predictive information than in what it costs to obtain that information and who is able to obtain it. CAC scoring has the strongest evidence base for reclassifying intermediate‐risk adults and is guideline‐endorsed but requires computed tomography, radiation exposure, and reporting expertise and is largely confined to urban tertiary centres in most LMICs. PRS offers a one‐off, lifetime‐stable measure at falling unit cost and has particular appeal in younger adults, in whom CAC is usually zero; however, its incremental value diminishes once clinical risk factors are known, it has not been shown to improve outcomes in randomised trials, and its poorer performance in non‐European ancestries is an equity concern rather than a technical detail [17, 18].

Inflammatory biomarkers occupy an intermediate position: hsCRP is inexpensive and widely available on existing laboratory platforms, but its incremental contribution to discrimination is small, and its clearest current use is in selecting patients for anti‐inflammatory therapy rather than in primary risk estimation [19, 20]. AI‐assisted tools are distinctive in that their marginal cost per patient is low once deployed, and they can extract risk information from data already being collected, such as electrocardiograms and retinal images; this makes them theoretically well‐suited to task‐shifted, primary care‐led screening. That potential is conditional on reliable digital infrastructure, external validation in the population of intended use and active surveillance for algorithmic bias, as models trained on unrepresentative data can systematically underestimate risk in the groups least well served by existing care [22].

Table 1 sets out this comparison across incremental predictive value, cost, infrastructure requirement, equity implications and implementation readiness. Figure 1 translates it into a stepwise pathway, in which universal, low‐cost assessment forms the base and selective second‐line testing is reserved for those in whom the result would change management and resource‐intensive approaches occupy a narrow apex. The pathway is intended to be read as tiered rather than sequential in every setting: A health system may reasonably implement only its lower tiers and still capture most of the achievable benefit.

## 5 Management Strategies

### 5.1 Pharmacological Interventions

Pharmacological management is fundamental to cardiovascular risk reduction, encompassing statins, antihypertensives and antidiabetic agents. Recent advances support the integration of traditional approaches with precision, patient‐centred strategies that emphasise both therapeutic intensification and, where appropriate, de‐escalation [44, 45].

#### 5.1.1 Statins

Statins remain central to primary and secondary prevention of CVD, substantially lowering LDL‐C and associated cardiovascular morbidity and mortality. Evidence confirms the safety and benefit of achieving very low LDL‐C levels, with intensive lowering reducing adverse cardiovascular outcomes without increasing non‐cardiovascular adverse events. In elderly patients and those with frailty, multiple comorbidities or limited life expectancy, discontinuing statins prescribed for primary prevention may be justified to reduce medication burden; statins should generally be continued for secondary prevention or in high‐risk patients [44].

#### 5.1.2 Antihypertensive Agents

Effective blood pressure control is another major pillar of CVD risk management. Guidelines recommend maintaining blood pressure below 140/90 mmHg, but individualised de‐escalation or withdrawal of therapy may be considered in selected patients, particularly younger individuals with low baseline pressure, on monotherapy and with sustained lifestyle modification. Successful withdrawal correlates with young age, lower starting blood pressure and the absence of significant comorbidity. For older patients or those at risk of orthostatic hypotension, cautious down‐titration is advised, with close monitoring for rebound and adverse events. Non‐pharmacological strategies, including weight loss, salt reduction and management of comorbidities such as obstructive sleep apnoea, support these efforts [44].

#### 5.1.3 Antidiabetic Agents

The antidiabetic treatment landscape includes a wide range of agents with distinct characteristics. Metformin remains first‐line therapy given effective glycaemic control, weight neutrality or modest loss and cardiovascular protection, though caution is required in elderly patients and in advanced renal impairment because of the risk of lactic acidosis and vitamin B12 deficiency [44]. Sulfonylureas and insulin are effective but increase hypoglycaemia risk, especially in older adults and in renal or hepatic impairment; dose reduction or substitution with lower risk agents, such as DPP‐4 inhibitors, is recommended in high‐risk groups [44, 45]. GLP‐1 receptor agonists and SGLT2 inhibitors offer glycaemic control together with cardiovascular benefit, weight reduction and minimal hypoglycaemia risk, though GLP‐1 receptor agonists may cause pancreatitis and SGLT2 inhibitors increase urinary and genital infection and, rarely, diabetic ketoacidosis; these agents should be discontinued if severe adverse effects occur [44, 45]. Periodic reassessment of glycaemic targets and treatment intensity is critical, particularly in frail individuals, those with limited life expectancy and patients who have recently experienced severe hypoglycaemia, in whom less intensive regimens are advisable [44]. Central to this approach is shared decision‐making that accounts for patient preferences, overall risk and comorbidity [45]. It should be acknowledged that access to the newer agents remains limited in many settings by cost, which constrains how far these recommendations can currently be applied globally.

### 5.2 Non‐Pharmacological Approaches (Lifestyle Modification)

Lifestyle modification is fundamental to cardiovascular risk reduction. A heart‐healthy dietary pattern, such as the Mediterranean or DASH diet, lowers LDL‐C, blood pressure and inflammation. Regular physical activity improves lipid profiles and insulin sensitivity, reduces inflammation and supports weight management. Smoking cessation reduces cardiovascular risk rapidly, whereas stress management through exercise, relaxation techniques and social support counters adverse metabolic effects. Evidence from clinical trials and guidelines demonstrates that these combined interventions produce significant and sustained reductions in cardiovascular risk and events [46]. These interventions also carry the lowest marginal cost of any strategy discussed in this review, and their delivery through primary care and community programmes is the intervention most likely to reduce population burden in the short term [47].

### 5.3 Precision Medicine in CVD Prevention

Precision medicine tailors interventions to individual genomic, proteomic, metabolomic and environmental profiles. The integration of multi‐omics technologies has facilitated deep phenotyping and enhanced risk stratification, allowing clinicians to predict, prevent and manage CVD with greater specificity. Key advances include the use of PRS to identify high‐risk individuals, genotype‐guided pharmacotherapy, novel biomarkers for early detection and AI‐assisted analysis of multi‐omic data. Despite implementation challenges relating to cost, access and data interpretation, precision strategies offer the prospect of earlier diagnosis, individualised therapy and fewer adverse effects, moving the field beyond population‐based algorithms toward patient‐centred preventive care [48]. A summary of pharmacological, non‐pharmacological and precision‐based strategies is presented in Table 3.

**TABLE 3:** Management strategies for cardiovascular disease prevention.

Category	Intervention	Key considerations
Pharmacological	Statins	Lower LDL‐C and reduce cardiovascular morbidity and mortality; intensive LDL‐C lowering is safe; deprescribing may be appropriate for frail elderly patients in primary prevention [44]
	Antihypertensives	Target blood pressure <140/90 mmHg; de‐escalation may be considered in selected low‐risk patients; monitor older adults for orthostatic hypotension; support with non‐pharmacological measures [44]
	Antidiabetic agents	Metformin is first‐line; sulfonylureas and insulin increase hypoglycaemia risk; GLP‐1 receptor agonists and SGLT2 inhibitors confer cardiovascular benefit; tailor therapy in frailty, renal impairment, or after adverse events; access is cost‐limited in many settings [44, 45]
Non‐pharmacological	Lifestyle modification	Mediterranean or DASH dietary pattern, physical activity, smoking cessation and stress reduction improve lipids, blood pressure, glycaemia and inflammation; strongly supported by trials and guidelines; lowest marginal cost of any strategy [46, 47]
Precision‐based	Omics‐based risk stratification	Genomics, proteomics, metabolomics and microbiomics enhance risk prediction and permit deep phenotyping; not yet outcome‐validated [48]
	Genotype‐guided pharmacotherapy	Tailors drug therapy to individual genetic profile (e.g., statin response and adverse‐effect susceptibility) [48]
	AI and multi‐omic data integration	AI tools analyse complex biological data to support individualised prevention; require validation in the intended population and surveillance for bias [22, 48]

Abbreviation: LDL‐C, low‐density lipoprotein cholesterol.

## 6 Challenges and Future Directions

The key challenges in combining conventional and novel approaches to cardiovascular risk assessment and management include limitations of traditional risk tools, underutilisation of novel biomarkers and imaging, disparities in implementation and insufficient system‐level integration. Inconsistent adoption of risk assessment tools, their poor integration into clinical workflows and inadequate attention to psychosocial determinants of health create persistent gaps in practice. The American Heart Association has emphasised that behavioural interventions and patient‐centred care models are least often implemented in underserved and under‐represented populations [47, 49, 50, 51]. Because Public Health Challenges addresses population‐level questions, the sections below consider these barriers in more detail.

### 6.1 Performance of Risk Tools in Diverse Populations

Most widely used risk equations were derived and validated in high‐income, predominantly European‐ancestry cohorts and systematically miscalibrated when applied elsewhere, over‐estimating risk in some populations and under‐estimating it in others [7, 9]. South Asian populations, which include that of Pakistan, develop coronary disease at younger ages and at lower BMI thresholds than the cohorts from which several equations were derived, so uncritical application may misclassify a substantial proportion of individuals. Region‐specific recalibration, as undertaken for the revised WHO charts and SCORE2, is a prerequisite for meaningful risk‐based care, and locally derived validation studies remain scarce in much of Africa, South Asia and the Middle East [10, 11].

### 6.2 Implementation in LMICs

The distribution of the CVD burden and the distribution of diagnostic capacity are inversely related. Advanced imaging, genomic testing and specialist cardiology services are concentrated in high‐income countries and in urban centres of middle‐income countries, whereas the majority of cardiovascular deaths occur outside them [29, 30]. In such settings the binding constraint is rarely the absence of a novel biomarker; more often it is intermittent availability of essential medicines, out‐of‐pocket payment, insufficient laboratory capacity for basic lipid and glucose testing and loss to follow‐up. The WHO HEARTS technical package was developed explicitly to address this implementation gap by standardising risk‐based management, treatment protocols, essential medicine supply, team‐based care and monitoring within primary care [4]. Emerging tools are most usefully evaluated by asking whether they can be layered onto such a platform, rather than by asking whether they outperform it.

### 6.3 Health System and Workforce Barriers

Even where technology is available, delivery depends on health system capacity. Shortages of trained personnel, the absence of structured referral pathways, fragmented records that prevent longitudinal follow‐up and reimbursement models that fund procedures rather than prevention all limit uptake. Task‐shifting to nurses, pharmacists and community health workers has been shown to improve risk factor control where physician density is low and is likely to be a more important determinant of population outcomes than the choice of risk marker. Any proposed pathway that presumes physician‐delivered assessment will therefore have limited reach in settings with the greatest need.

### 6.4 Digital Health Infrastructure and Governance of AI

AI‐assisted risk assessment depends on digital infrastructure that is unevenly distributed: reliable electricity, connectivity, interoperable electronic records and local technical support. Where these exist, AI applied to routinely collected data offers a plausible route to extending risk assessment at low marginal cost. Where they do not, promoting AI‐based tools risks diverting attention from more tractable priorities. Governance is an equally substantive concern. Models trained on unrepresentative datasets can perform poorly in the groups least well served by existing care, and bias may be introduced at any stage from problem formulation through post‐deployment monitoring [22]. Prospective external validation in the intended population, reporting of subgroup performance and mechanisms for ongoing audit should be regarded as minimum conditions for clinical deployment rather than as refinements.

### 6.5 Clinician Adoption and Integration Into Clinical Workflow

Risk assessment tools are frequently underused even where freely available, reflecting time pressure in consultations, uncertainty about how to act on discordant results, limited familiarity with newer markers and the absence of decision support embedded in the record system. A CAC score or PRS result that arrives without an accompanying management pathway is unlikely to change practice. Integration into existing workflows, clear thresholds for action, and clinician education are therefore not secondary implementation details but determinants of whether any of these tools produce benefit.

### 6.6 Equity of Access to Advanced Testing

If advanced testing is adopted before questions of access are settled, the predictable consequence is that those already best served will benefit first, widening the outcome gap that risk stratification is intended to close. This applies with particular force to PRS, whose accuracy currently depends on ancestry [18], and to CAC scoring, whose cost is typically borne out of pocket in systems without universal coverage. Equity considerations argue for prioritising tools that are cheap, calculable from data already collected and deliverable in primary care, and for treating advanced testing as an adjunct for defined clinical uncertainty rather than as a general standard of care.

### 6.7 Patient‐Centred Care and Shared Decision‐Making

Risk communication is itself an intervention. Numerical risk estimates are frequently misunderstood, and the addition of further tests may increase anxiety without altering management. Person‐centred models that elicit patient values, address health literacy and support shared decision‐making improve adherence and are endorsed in current statements [49, 50]. Their absence is most pronounced in the populations at highest risk, which is itself an equity issue.

### 6.8 Research Priorities

Several priorities follow from the preceding discussion. First, randomised evidence is needed on whether risk assessment strategies incorporating CAC, PRS or AI‐assisted tools improve clinical outcomes, rather than only reclassification metrics. Second, derivation and validation cohorts must be diversified, with genomic and clinical datasets from South Asian, African and Middle Eastern populations. Third, cost‐effectiveness analyses should be conducted in the health systems where the tools would actually be deployed, not extrapolated from high‐income settings. Fourth, implementation research is required on task‐shifting, digital delivery and integration with existing NCD programmes. Fifth, reporting standards for AI‐based cardiovascular tools should require subgroup performance data as a condition of publication and regulatory approval.

## 7 Conclusion

The evolving landscape of cardiovascular risk assessment and management supports a shift from uniform, one‐size‐fits‐all models toward more individualised approaches. Conventional risk factors remain foundational and continue to account for the majority of population attributable risk, but they do not capture the full spectrum of cardiovascular vulnerability, particularly in diverse and intermediate‐risk populations. Emerging tools—inflammatory and genetic biomarkers, advanced imaging and lifestyle and environmental risk modifiers—provide incremental value in refining prediction and guiding tailored prevention, though the magnitude of that value is generally modest and its translation into improved outcomes is not yet established. Precision medicine, AI‐assisted analytics and patient‐centred care offer plausible routes to earlier detection and better outcomes, but their public health contribution will be determined by cost, infrastructure and equity of access rather than by discriminative performance alone. A tiered approach, in which universal low‐cost assessment is delivered reliably and advanced testing is reserved for defined clinical uncertainty, is likely to yield greater population benefit in the near term than broad adoption of novel tests. Addressing the global burden of CVD more effectively and equitably will require that innovation in risk prediction be matched by investment in the systems through which prevention is actually delivered.

## Author Contributions

**Azka Mujeeb** writing – original draft. **Ahmed Asad Raza**: supervision, writing – original draft, writing – review and editing. **Abedin Samadi**: writing – review and editing. **Sana Rasheed**: conceptualisation, writing – original draft, writing – review and editing, resources. **Misbah Sarfraz**: writing – original draft, writing – review and editing.

## Funding

The authors have nothing to report.

## Ethics Statement

The authors have nothing to report.

## Consent

The authors have nothing to report.

## Conflicts of Interest

The authors declare no conflicts of interest.

## Data Availability

No datasets were generated or analysed during the current study.
